# Bioevaluation and Targeted Modification of Temporin-FL From the Skin Secretion of Dark-Spotted Frog (*Pelophylax nigromaculatus*)

**DOI:** 10.3389/fmolb.2021.707013

**Published:** 2021-10-19

**Authors:** Wenjie Wang, Wanqing Yang, Shouying Du, Xinping Xi, Chengbang Ma, Lei Wang, Mei Zhou, Tianbao Chen

**Affiliations:** ^1^ School of Chinese Materia Medica, Beijing University of Chinese Medicine, Beijing, China; ^2^ Natural Drug Discovery Group, School of Pharmacy, Queen’s University Belfast, Belfast, United Kingdom

**Keywords:** amphibian skin, anti-biofilm, antimicrobial peptide, peptide modification, temporin

## Abstract

Bioactive proteins secreted by the granular glands of amphibian skin play a self-defensive role, and exhibit various bioactivities *in vitro* and *in vivo*. In light of the severity of the problem of antibiotic resistance for treating infections, many antimicrobial peptides (AMPs) have been developed and applied in clinical microbial treatments. We identified a naturally derived and potent antimicrobial peptide, temporin-FL, obtained from the skin secretion of *Pelophylax nigromaculatus* via “shotgun” cloning. Two truncated analogues of this peptide were chemically synthesized to explore their structural-functional relationships. The results of a functional evaluation showed that all of the tested AMPs were active against Gram-positive bacteria and fungi and demonstrated antibiofilm activity against methicillin-resistant *Staphylococcus aureus* (MRSA) but did not have an effect on Gram-negative bacteria. Moreover, temporin-FLa demonstrated a higher level of hydrophobicity and enhanced antimicrobial efficiency, as well as hemolytic activity and cell cytotoxicity than the parent peptide. Temporin-FLb, which evidenced significantly less α-helicity, was less potent against various microbes but exhibited lower cytotoxicity relating to mammalian cells. Both of the synthesized analogues possessed a higher therapeutic index than the original peptide. Moreover, the membrane permeability assay and the measuring membrane depolarization assay declared that temporin-FL and its analogues induced membrane fracture and depolarization; the quantitative biofilm formation assay and the observations of MRSA biofilms using scanning electron microscopy revealed that the AMPs caused biofilm disruption and blocked biofilm formation, the former experiments all confirming their antimicrobial and antibiofilm properties. Hence, the optimization of temporin-FL offers insights for the discovery of new drugs for treating MRSA infections.

## Introduction

The prevalence of microbial infections poses a major threat to public health, with antibiotics being the predominant treatment against pathogenic microorganisms. However, the abuse of antibiotics has led to the emergence of multiple drug-resistant bacteria, including Methicillin-resistant *Staphylococcus aureus* (MRSA). Research has shown that MRSA strains generated from adaptable *Staphylococcus aureus* (*S. aureus*) have developed resistance to methicillin via the *mecA* gene mutation (Chambers and Deleo, 2009). Moreover, these strains developed multidrug resistance rapidly via a horizontal gene transfer and chromosomal mutation, making MRSA the greatest pathogenic threat to human health (Craft et al., 2019). Meanwhile, MRSA was able to spontaneous aggregate and form an organized population called biofilm, which exhibited higher resistance in a harsh environment by forming the extracellular matrix to protect inner bacteria from damage and remaining slow-growing state to limit antibiotic efficacy (Craft et al., 2019).

Because the continual emergence of antibiotic-resistant microbes is associated with elevated lethality rates, efficient anti-infection drugs are urgently required. The skin of amphibians is a critical component of their self-defending system, as it secretes various bioactive compounds against pathogens (Conlon, 2004), adjusts blood flow, and stimulates water absorption (Hillyard and Willumsen, 2011). Antimicrobial peptides (AMPs) isolated from frog skin secretion are considered the most efficient substances for immune defence. Under conditions of stimulation or injury, the granular glands of amphibians release high concentrations of bioactive peptides in a holocrine manner (Romero et al., 2020). AMPs have been shown to possess strong capabilities against bacteria, fungi, viruses, and even cancer cells that are associated with non-specific mechanisms (Ageitos et al., 2017; Sierra et al., 2017). In addition, in a context of widespread concern relating to multi-drug resistant bacterial infections, AMPs have been proven to be superior to conventional antibiotics given their broad-spectrum antimicrobial activity that does not induce resistance, especially against multi-antibiotic-resistant bacteria. Consequently, AMPs are promising candidates for antimicrobial drug development.

Ten distinct temporins were first identified from the skin secretion of the European red frog, *Rana temporaria* (Simmaco et al., 1996). Short amino acid sequences (10–14 amino acids), net positive charge (ranging from 0 to +3), and α-helical structure are characteristic traits of the temporin family (Mangoni, 2006; Romero et al., 2020). Temporins lack the “Rana-box” motif comparing with other AMP families from Ranaidae peptides, but the C-terminus of temporin precursor sequences possesses a highly conserved region, which results in the post-translation modification of C-terminal amidation of mature peptide (Simmaco et al., 1996; Mangoni, 2006). Temporins demonstrate a broad-spectrum inhibitive effect against most pathogens and lower levels of cell toxicity. They have a positive net charge in a neutral pH resulting from basic amino acid residues (generally Lysine) and terminal amidation. Previous studies have found that the mechanism of antimicrobial activity is associated with penetration of the microbial phospholipid bilayer, with antimicrobial efficacy being positively correlated with the net charge of temporins, as temporins with a lower charge are insensitive toward most microbes (Simmaco et al., 1996; Romero et al., 2020). Apart from the net charge of temporins, their amino acids sequence also affects their bioactivities via different mechanisms, with the content of hydrophobic residue altering the hydrophobicity and amphipathicity of temporins, which alters AMP-membrane affinity and affects membrane-perturbing potency (Mangoni et al., 2000). The peptide-membrane interactions were focused on the study of peptide antimicrobial mechanisms, to form a transmembrane pore, the peptides were aggregated on the membrane surface (Travkova et al., 2017). In addition, the peptide-membrane interactions were mainly driven by electrostatic binding, while the hydrogen bond and hydrophobic interaction also play important roles in peptide aggregation (Travkova et al., 2017). The structural-functional relationships of truncated peptide derivatives have emerged as a hot research topic in AMP modification studies, intending to enhance the bioactive efficiency of AMPs for broader adaptiveness in different pathogens while lowering the costs incurred in chemical synthesis. In particular, previous researches demonstrated that AMPs in the Rana genus including ranatuerin-2Pb and brevinin-2GHk, their truncated analogue showed improved bioactivity (Zhou et al., 2019; Chen et al., 2020). Therefore, peptide truncation is a positive modification strategy for optimizing selectivity and bioactivity.

In this study, the nucleotide sequence of prepropeptide cDNA was identified through “Shotgun” cloning, then the mature AMP named temporin-FL was isolated from *Pelophylax nigromaculatus* using reverse-phase high-performance liquid chromatography (RP-HPLC) and mass-spectrum techniques. To conduct structural-functional studies, the native peptide and its truncated derivatives, temporin-FLa and temporin-FLb, were chemically synthesized via the solid-phase peptide synthesis system (SPPS). A structural analysis was conducted using online analytical tools and the circular dichroism spectrum. Antimicrobial, antibiofilm, hemolytic and cell viability assays were performed to evaluate the biological activities and toxicity of the three AMPs. They were subsequently subjected to membrane-permeability and depolarization assay to determine the mechanism of AMP’s antimicrobial activities. Then the peptides in different concentrations were conducted quantitative biofilm formation assay and the biofilm was observed using scanning electron microscopy (SEM) to evaluate their effect on MRSA biofilm formation and integrity.

## Materials and Methods

### Acquisition of Skin Secretion From *Pelophylax nigromaculatus*

Adult specimens of *Pelophylax nigromaculatus* (dark-spotted frog) were purchased from commercial sources in China. The skin secretions were harvested from the dorsal skin of the frogs via gentle transdermal electrical stimulation. The skin secretions were then rinsed by distilled deionized water and collected, and the solution was snap-frozen using liquid nitrogen before being lyophilized following the procedure as previously described (Chen et al., 2016). The acquisition process was conducted according to the guidelines in the United Kingdom Animal Scientific Procedures and was performed under appropriate United Kingdom animal research licenses.

### Identification of Precursor-Encoding cDNA From Skin Secretion via “Shotgun” Cloning

The “shotgun” cloning was carried out as previously described (Chen et al., 2016). Briefly, an aliquot of 5 mg of lyophilized skin secretion was weighed and dissolved in 1 ml lysis/binding buffer (DYNAL Biotech, United Kingdom). The mRNA in the skin secretion was isolated using the Dynabeads^®^ mRNA DIRECT™ Kit (DYNAL Biotech, United Kingdom) and a cDNA library was constructed using the SMART-RACE kit (BD Bioscience Clontech, United Kingdom). A degenerated primer (5′- GAWYYAYYHRAGCCYAAADATG-3′) (W = A/T; Y = C/T; H = A/C/T; R = A/G; D = A/G/T) used in the 3′RACE procedure was designed based on a highly conserved domain within the 5′ untranslated regions of closely-related *Rana* species. The polymerase chain reaction products were purified using the pGEM^®^-T Easy Vector (Promega Corporation, Southampton, United Kingdom), and an ABI 3100 automated capillary sequencer (Applied Biosystems, Foster City, CA, United States) was subsequently used to analyze the nucleotide sequences of purified DNA fragments. The nucleotide and amino acid sequence analyses were carried out using the NCBI-BLAST tool.

### Isolation and Identification of Peptide From the Skin Secretions

To confirm the primary structure of naturally produced peptide from skin secretion, the isolation and identification of peptide from lyophilized skin secretion were conducted as the former study described (Liu et al., 2017). The skin secretion (5 mg) was dissolved in 1 ml of water/trifluoracetic acid (TFA) (99.95/0.05, v/v) and centrifuged to obtain the supernatant. The obtained solution was then isolated using a Cecil CE4200 Adept gradient RP-HPLC system (Cecil, Cambridge, United Kingdom) with a C-18 column (250 mm × 21.2 mm, 5 μm, 300 Å; Phenomenex, United Kingdom). The flow rate was 1 ml/min with gradient elution from TFA/water (0.05/99.95, v/v) to TFA/water/acetonitrile (0.05/19.95/80.00, v/v/v) in total 240 min, the fractions were collected as 1-min interval. MALDI-TOF mass spectrometry (Voyager DE, PerSeptive Biosystems, Framingham, MA, United States) was performed to analyze the mass-to-charge ratio of the collected fractions, and the positive detection matrix was α-cyano-4-hydroxycinnamic acid (CHCA). The presumed target fraction was presented to the LCQ-Fleet electrospray ion-trap mass spectrometer (Thermo Fisher Scientific, San Francisco, CA, United States), and MS/MS fragmentation sequencing was performed.

### Design and Chemical Synthesis of Peptides

The helical structure of AMPs and their physicochemical properties, notably amphipathicity and hydrophobicity, have crucial effects on their bioactivity. To investigate the structural-functional relationship of AMPs, we used the Fmoc solid-phase peptide synthesis system (Protein Technologies, Tucson, AZ, United States) to synthesize temporin-FL (FIPLVSGLFSKLL-NH_2_) and its two truncated analogues: temporin-FLa (FIPLVSGLFKLL-NH_2_, remove Ser^10^) and temporin-FLb (FIPVSGLFKLL-NH_2_, remove Leu^4^ and Ser^10^). The antimicrobial peptide prediction website CAMPr3 (http://www.camp.bicnirrh.res.in/index.php) was applied to calculate the antimicrobial probability of truncated peptides in two different models. The chemically produced analogues were then purified using the RP-HPLC system, and the fractions containing the targeted peptides were confirmed by obtaining their molecular mass using MALDI-TOF mass spectrometry (Voyager DE, PerSeptive Biosystems, Framingham, MA, United States).

### Conformation Studies

The physiochemical structural analysis entailed website prediction and circular-dichroism (CD) spectroscopy as described previously (Liu et al., 2017; Sang et al., 2018; Zhou et al., 2019). Briefly, temporin-FL and two analogues were dissolved in 10 mM NH_4_Ac or 10 mM NH_4_Ac with 50% 2,2,2-trifluoroethanol (TFE) to determine their conformation in different solutions. The 10 mM NH_4_Ac solution provides a pH stable buffering environment to stabilize peptides and also to prevent system noise and not interfere with CD signals. The results of the CD spectroscopy were inputted into the BESTSEL CD spectrum online analysis tool (http://bestsel.elte.hu/index.php). HeliQuest (https://heliquest.ipmc.cnrs.fr/), and I-Tasser (http://zhanglab.ccmb.med.umich.edu/I-TASSER) were respectively used to determine the helical wheels plots and to predict the 3D secondary structure.

### Antimicrobial Activity

Antimicrobial activity was evaluated by assessing minimal inhibitory concentrations (MICs) and minimal bactericidal concentrations (MBCs). The tested microorganisms comprised three Gram-positive bacterial strains, namely *S. aureus* (NCTC 10788) and MRSA strains (NCTC 12493 and ATCC 43300), *E. coli* (NCTC 10418), which is a Gram-negative bacterial strain, and *C. albicans* (NCYC 1467), a yeast species. Standard microbes were cultured in Muller-Hinton Broth (MHB) and suspended to obtain 5 × 10^5^ CFU/ml. Subsequently, the equal volume microorganisms solutions were treated with a 2-fold dilution of peptides, the final concentration of the peptides ranged between 512 and 1 μM. The positive control of bacteria strains *S. aureus*, MRSA NCTC 12493 and *E. coli* is 0.2 mg/ml norfloxacin, 16 μM mellitin was used as *C. albicans* positive control and 0.2 mg/ml vancomycin as a positive control in ATCC 43300 MRSA strains. The solutions were incubated in 96-well plates used for measuring MICs followed for 18 h. The MBC values were assessed based on observations of the colony cultured on Muller-Hinton agar (MHA). The antimicrobial assay was performed in triplicate following the procedure applied in previous studies described (Huang et al., 2017).

### Antibiofilm Activity of the Three AMPs Determined From the MRSA Biofilm

We evaluated the antibiofilm activity of the three AMPs by applying a crystal violet staining assay following previous research with minor modifications (Chen et al., 2018), to determine the minimal biofilm inhibitory concentrations (MBICs) and the minimal biofilm eradication concentrations (MBECs). The tested MRSA bacterial strains (NCTC 12493 and ATCC 43300) were incubated and diluted with MHB to achieve a bacterial concentration of 5 × 10^5^ CFU/ml. The peptides were undergone a 2-fold dilution and were incubated with diluted MRSA to determine MBIC values. For the MBEC assay, the diluted bacterial culture was incubated for 48 h to obtain a fully grown biofilm, which was treated with various concentrations of peptides dissolved in MHB and incubated for 24 h at 37°C. The positive control in MBIC and MBEC determination is 0.2 mg/ml vancomycin for MRSA ATCC 43300 strain and the aliquote of norfloxacin for MRSA NCTC 12493.

### Quantitative Biofilm Formation Assay

Based on the previous study of quantitative biofilm formation assay (Badmasti et al., 2015; Liu et al., 2020), the crystal violet staining method with moderate modifications was applied for analyzing the capability of MRSA (ATCC 43300) forming biofilms when treated with different concentrations of peptides. Specifically, MRSA strain was cultured in MHB medium at 37°C overnight. Then the bacterial culture was subcultured in fresh MHB medium. The quantificational bacterial suspension (5 × 10^5^ CFU/ml) was added into a 96-wells plate together with the tested peptides at different concentrations (2 × MBIC, 1 × MBIC, 1/2 × MBIC and 1/4 × MBIC). The MHB medium without bacteria was negative control and MRSA culture solution was the positive control. The cut-off optical density (OD_c_) was the mean ± deviation value of the absorbance of negative control at 595 nm. The biofilm formation ability of bacteria samples with different concentrations of peptides were divided into four groups: the negative biofilm formation (−) was the OD_595nm_ ≤ OD_c_, the weak biofilm formation (+) was the OD_c_ < OD_595nm_ ≤ 2 × OD_c_, the moderate biofilm formation (++) represented the 2 × OD_c_ < OD_595nm_ ≤ 4 × OD_c_, and the strong biofilm formation (+++) indicated the OD_595nm_ > 4 × OD_c_.

### Observation of MRSA Biofilms and the Biofilm Disrupting Potency of Synthesized Peptides Based on Scanning Electron Microscopy

Silicon wafers were cut into small squares (5 mm × 5 mm) and placed at the bottom of a 24-well plate. Each well was then filled with 1 ml of sub-cultured MRSA (ATCC 43300) in MHB at a concentration of 5 × 10^5^ CFU/ml and incubated at 37°C in a humid shaking incubator for 48 h to enable the formation of visible biofilms on the silicon wafers. Peptides at concentrations of 1 × MBEC and 4 × MBEC were incubated with PBS-washed MRSA biofilm for 24 h. After the incubation, the silicon wafer in each well was first washed in PBS and then fixed in 2.5% glutaraldehyde at 4°C for 4 h. The fixed biofilm was dehydrated using gradient ethanol solution (30, 50, 70, 80, 90, 95, and 100%) at 10-min intervals. The air-dried biofilm samples were coated with gold film using a LEICA-EM-ACE600 sputter coater instrument (Leica, Germany) and transferred to the field emission scanning electron microscopy (FESEM) system (ZEISS-SUPRA55, Germany) operating at 5 kV to observe the morphology of the peptide-treated biofilm.

### Membrane Permeability Assay

The bactericidal activity of cationic peptides resulted from membrane disruption. DNA or RNA that leaked out of the cell was stained with SYTOX^™^ Green Nucleic Acid Stain (Invitrogen Life Technologies, Carlsbad, CA, United States). The MRSA ATCC 43300 bacterial strain was used to evaluate the membrane disruptive potency of the three peptides. The assay was performed according to the procedure described previously with some modifications (Chen et al., 2018). Peptides at different concentrations (4 × MIC, 2 × MIC and 1 × MIC) were incubated with the re-suspended bacterial solution at a concentration of 5 × 10^6^ CFU/well in the 96-well black plate for 3 h without light. The positive control is 70% isopropanol. SYTOX green dye (the final concentration of 1%) was then added to each well, and the permeabilizing potency was measured as fluorescent intensity using a SpeactraMax i3x plate reader (Molecular Devices, CA, United States). All experiments were done in five replicates for statistical significance.

### Membrane Depolarization Assay

The membrane depolarization assay was conducted by Chakraborty’s method with some modifications (Chakraborty et al., 2012). The MRSA ATCC 43300 strain was cultured in MHB medium to the exponential phase (OD_550nm_ = 0.20). Then the bacterial suspension was diluted until the final bacterial density was 10^8^ CFU/ml, afterwards, the bacterial suspension was added into clean centrifuge tubes (5 ml/tube). The bacterial precipitate was harvested by centrifugation (4,500 rpm, 5 min at 4°C), then washed twice by phosphate-buffered saline. The bacteria were re-suspended by fresh MHB with different concentrations of peptides (the final concentrations of 4 × MIC, 1 × MIC and 1/4 × MIC) and incubated in a shaking incubator at 37°C for 4 h. The MRSA bacterial culture was regarded as the control group. After incubation, 90 μL of bacterial culture was transferred into black 96-wells plate together with 10 μL of rhodamine 123 fluorescent dye (the final concentration of 5 μg/ml) (Solarbio, Beijing, China). The plate was incubated in the dark for 15 min to allow the dye incorporate into bacteria. The Rh123 fluorescence was monitored by a SpeactraMax i3x plate reader (Molecular Devices, CA, United States) with the excitation and emission wavelengths of 488 and 530 nm, respectively.

### Hemolytic Assay

Following a previous study (Huang et al., 2017), we evaluated the hemolytic activity of three peptides using defibrinated erythrocytes isolated from whole horse blood (TCS Biosciences Ltd., Buckingham, United Kingdom). Phosphate-buffered saline (PBS) and 1% Triton X-100 were used as negative and positive controls, respectively, and 1% DMSO/PBS was used as the vehicle control. The HC_50_ and the therapeutic index (TI = HC_50_/the geometric mean of MICs against tested bacterial strains of peptide) were calculated to determine the therapeutic potential.

### Cell Viability Assay

Human keratinocyte cell line HaCaT (No.1101HUM-PUMC000373) was obtained from the National Infrastructure of Cell Line Resource (Beijing, China) and cultured in high-glucose Dulbecco’s modified Eagle medium (DMEM), containing 10% fetal bovine serum (FBS) and 1% penicillin and streptomycin (Thermo Fisher Scientific Co., Waltham, MA, United States). The grown cells were digested and quantificationally harvested in a 96-wells plate of cell density of 5 × 10^4^×cells/ml. After 24 h incubation, the original medium in wells was replaced with fresh medium containing peptides with 2-fold serial dilution, the final concentrations of peptides ranging from 512 Μm to 1 μM. When the cells were treated with peptides for 24 h, the cell medium was changed into 100 μl of 10% CCK-8 solution and incubated for 4 h. The absorbance at 450 nm reflected the cell viability in each well. The growth control was cell culture without peptides and blank control was fresh DMEM without cells.

### Statistical Analysis

The data analysis was performed with the Prism 6.0 software (GraphPad Software Inc., San Diego, CA, United States). The error bar indicated the standard error of the mean (SEM) of more than three replicates in the different experiments.

## Results

### Identification and Characterization of Precursor-Encoding cDNA of Temporin-FL From the Skin Secretion of Frogs

**FIGURE 1:**
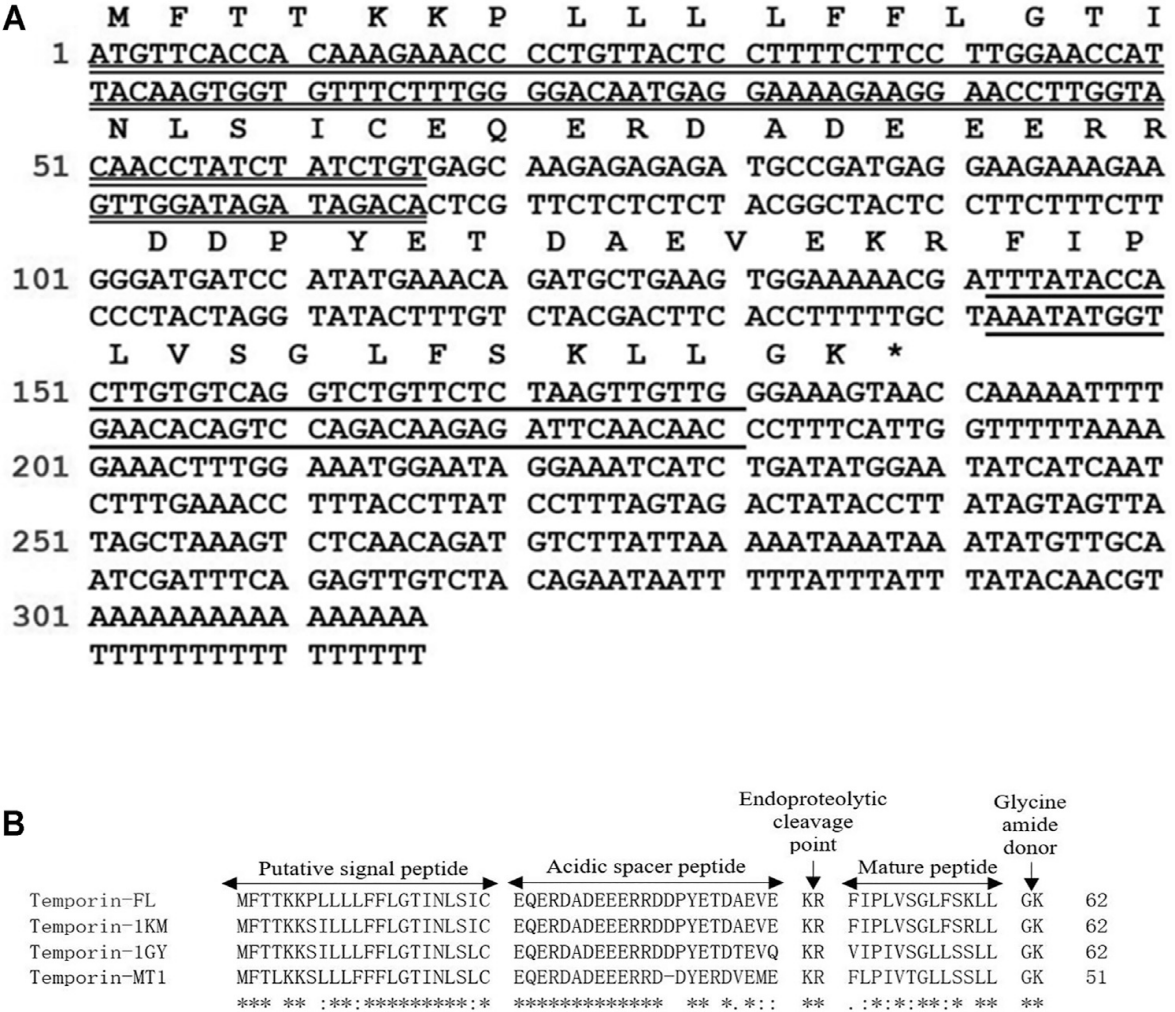
Nucleotide and translated Open Reading Frame (ORF) amino acids sequences of the precursor cDNA encoding the peptide temporin-FL from the skin secretion of *Pelophylax nigromaculatus*. The putative signal peptide sequence is double-underlined, the mature peptide sequence is single-underlined. The asterisk indicates the stop codon **(A)**. Alignments of the amino acid sequences of the prepropeptides of temporin-FL, temporin-1KM, temporin-1GY and temporin-MT1 comprise five domains. The identical amino acids are indicated by asterisks **(B)**.

We analyzed the nucleotide sequence (316 base pairs) of the open reading frame (ORF) of the precursor cDNA encoding the prepropeptide temporin-FL from powdered skin secretions of *Pelophylax nigromaculatus*. The analysis revealed a signal peptide region (23 amino acid residues) and an acidic spacer ending with a typical Lys-Arg-propeptide convertase processing site (Figure 1A) within the amino acid sequence of the ORF. The mature peptide sequence of temporin-FL contained 13 amino acid residues and a C-terminal-Gly-Lys- region that served as an amidating domain for C-terminal amidation of the mature peptide. The findings of NCBI-BLAST indicate that temporin-FL has three similar peptides. Alignment of the ORF-translated amino acid sequences of the novel peptide and three similar peptides, namely temporin-1KM (*Pelophylax nigromaculatus*), temporin-1GY (*Pelophylax nigromaculatus*) and temporin-HB3 (*Pelophylax hubeiensis*) showed a remarkably high degree of primary structural similarity of their amino acid sequences. This finding suggests that the mature peptide belongs to the temporin family according to the highly conserved putative N-terminal peptide region (Figure 1B). The nucleotide sequence data of prepropeptide temporin-FL was registered in the GenBank Nucleotide Sequence Database, No. MT354841.

### Identification and Verification of Temporin-FL From the Skin Secretion of Frogs

**FIGURE 2:**
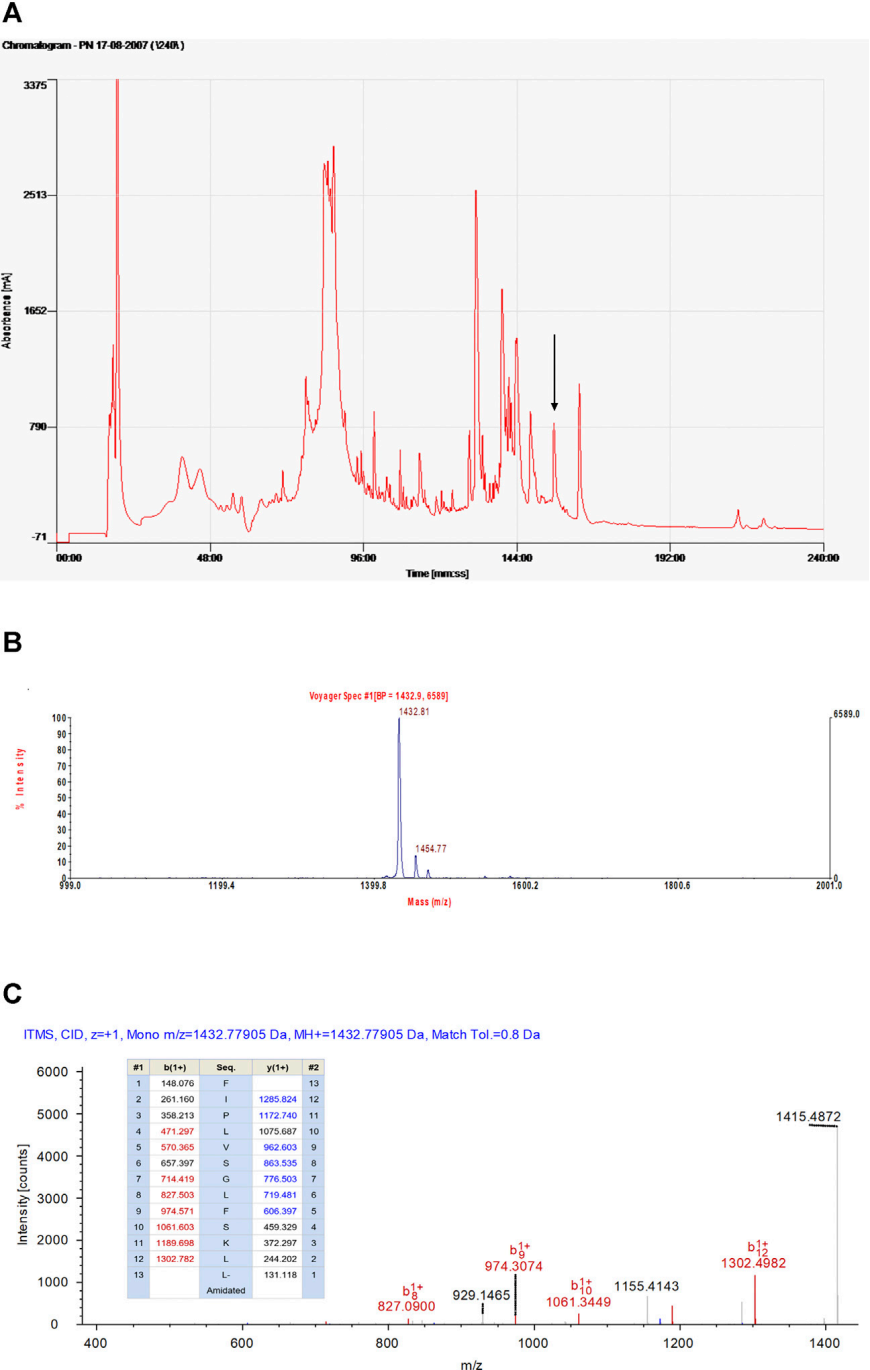
The RP-HPLC chromatogram of *Pelophylax nigromaculatus* skin secretion indicating the elution peak (showed by arrow) and retention time of temporin-FL in 156 min **(A)**. The mass spectra of fraction in 156 min corresponding to temporin-FL, the mass-to-charge ratio is 1,432.81 **(B)**. The annotated tandem mass (MS/MS) fragmentation spectra and predicted singly-charged b-ions and y-ions arising from MS/MS fragmentation of temporin-FL. The coloured typeface indicating the actual fragment **(C)**.

RP-HPLC, MALDI-TOF mass spectrometry, and LCQ-Fleet electrospray ion-trap mass spectrometer were used to identify and verify temporin-FL (the actual molecular mass is 1,432.78) obtained from frog skin secretion. The retention time of temporin-FL in the RP-HPLC system was 156 min, and the molecular mass of the elution fraction was 1,432.81 Da in MALDI-TOF mass spectrogram. The amino acid sequence was determined by performing MS/MS fragmentation sequencing, the results of which are shown in Figure 2.

### Peptide Design and Conformational Studies

**FIGURE 3:**
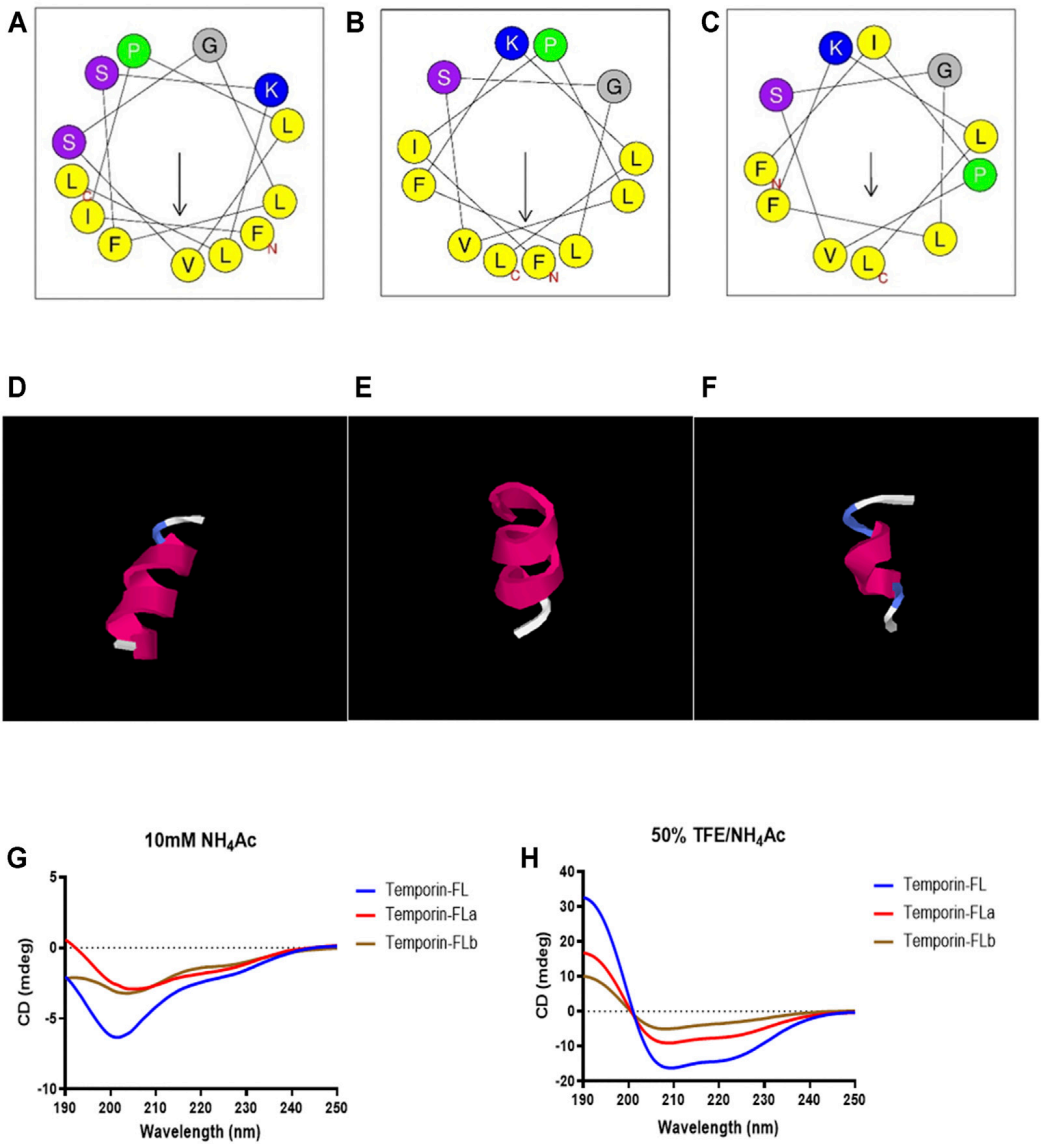
The helical wheels of temporin-FL **(A)** temporin-FLa **(B)** and temporin-FLb **(C)**; the secondary structure prediction of temporin-FL **(D)** temporin-FLa **(E)** and temporin-FLb **(F)**; CD spectrogram of three peptides in 10 mM ammonium acetate water solution **(G)** and 50% TFE/10 mM ammonium acetate water solution **(H)**.

The results of conformational studies performed with an online analytical tool are shown in Figure 3 and Table 1. The helical wheel plots and prediction of the 3D secondary structure indicated that the three peptides have different hydrophobic faces and helical patterns. The small temporins are inactive against most bacterial strains (Simmaco et al., 1996), however, truncation of amino acids is widely applied for adjusting peptide physicochemical properties, including membrane affinity (Feng et al., 2020; He et al., 2020; Zhu et al., 2020). According to previous studies, modification of temporins is always performed via amino acids alteration or terminal sequence addition (Mangoni et al., 2011; Bezzerri et al., 2014; Xie et al., 2019), the rational truncation of selected amino acid residues is quite rare. In this study, two novel peptide derivatives were intentionally designed to investigate how the truncation of non-charged amino acids affects the bioactivity of small peptides. Removing one of two polar residues would highly enhance its hydrophobicity and hydrophobic moment. Comparing with the sixth serine residue truncation, the truncated modification of removing Ser^10^ from the hydrophilic face made temporin-FLa possess an increased hydrophobic moment (0.662), and the antimicrobial probability score also higher than the sixth serine residue truncation in the Support Vector Machines model. According to previous studies, the increased amphipathicity probably leads to a strong hemolytic effect, to investigate the relationship between membrane selectivity and peptide amphipathicity, we designed the analogue temporin-FLb, an N-terminal truncated peptide based on temporin-FLa with a lower hydrophobic moment score (0.394) and approximately the same hydrophobicity value (1.035) as the parent peptide. Temporin-FLb has the lowest hydrophobic moment value than other N-terminal truncated mutations, and the AMP probability score in the two models also exceed 0.9, indicated that temporin-FLb has a strong possibility to become an antimicrobial peptide, while the derivative 6 with the high score of hydrophobicity was not considered as ideal derivates, for high hydrophobicity may affect the bioactivity evaluation (The comparison of the parent peptide and probable derivatives were shown in Supplementary Figure S3 and Supplementary Table S1). The synthesized peptides presented a predominant α-helix structure in 50% TFE/NH_4_Ac solution, as indicated by the secondary structure accessed via the CD spectrum. However, the helicity was eliminated in the NH_4_Ac solution. Our findings indicate that the native peptide exhibits the highest helical percentage (92.3%), with the percentage declining in the two analogues as a result of the reduction in amino acids (58.7 and 31.9%, respectively).

**TABLE 1:** The primary, secondary structures and physicochemical properties of temporin-FL, temporin-FLa and temporin-FLb, the truncated position are underlined.

	Peptides		
	Temporin-FL	Temporin-FLa	Temporin-FLb
Sequence	FIPLVSGLFSKLL-NH_2_	FIPLVSGLF_KLL-NH_2_	FIP_VSGLF_KLL-NH_2_
Structural prediction^a^	CCCHHHHHHHHHC	CCCHHHHHHHHC	CCCCCHHHHCC
Confident score	9314888888549	920476687649	94500445309
AMP probability in SVM model	0.961	0.966	0.923
AMP probability in RF model	0.967	0.97	0.9005
Hydrophobicity (H)	1.004	1.091	1.035
Hydrophobic moment (μH)	0.553	0.662	0.394
Net charge	+2	+2	+2
Non-polar residues (%)	69.23	75	72.73
Helix in 50%TFE/NH_4_Ac (%)	92.3	58.7	31.9
Helix in NH_4_Ac (%)	16.7	11.0	17.2

a C, Coli, H, Helix, SVM, Support Vector Machines, RF, Random Forests.

### An Evaluation of the Therapeutic Effect and Cytotoxicity of Synthetic Peptides

As shown by the results presented in Table 2 and Supplementary Figure S1, at MICs of 16–32 μM, the native peptide, temporin-FL, demonstrated strong activity against tested Gram-positive bacterial strains and fungi but was inactive against Gram-negative bacteria *E. coli*. Temporin-FLa was more potent than other peptides with four times lower MICs than temporin-FL against Gram-positive bacteria and fungi. Temporin-FLb, which is the shortest peptide analogue, exhibited relative weak antimicrobial activity. The MBC values represent the minimum bacterial eradicated concentration, which was about two to four times higher than the MICs for AMPs. By contrast, temporin-FLa exhibited stronger bactericidal potency against antibiotic-resistant strains.

**TABLE 2:** The antimicrobial and antibiofilm activities against tested bacteria strains and cell cytotoxicity.

		Temporin-FL	Temporin-FLa	Temporin-FLb
Antimicrobial activity MIC/MBC (μM)	*S. aureus*	16/16	4/8	8/16
	MRSA NCTC 12493	32/64	4/16	16/32
	MRSA ATCC 43300	32/32	4/8	64/128
	*E. coli*	128/128	128/128	128/128
	*C. albicans*	32/64	8/32	32/128
Antibiofilm activity MBIC/MBEC (μM)	MRSA NCTC 12493	32/64	8/32	64/128
	MRSA ATCC 43300	32/64	4/8	64/128
Biofilm formation (2×, 1×, 1/2×, 1/4×MBIC)	MRSA ATCC 43300	NE/NE/++/++	NE/NE/++/+++	NE/NE/++/+++
Hemolysis effect	HC_50_	157.4	74.02	356.0
Cell cytotoxicity	IC_50_	83.38	46.62	375.1
Therapeutic index (TI)	TI (MRSA)	4.92	18.51	11.13
	TI (Overall)	4.28	8.05	11.13

The MBIC represented the minimum biofilm inhibitory concentration that impeded initial bacterial attachment for further biofilm formation, as shown in Table 2. The MBIC values of temporin-FL were equal to those of MICs for different MRSA strains, and the MBIC value of temporin-FL against MRSA ATCC 43300 was the same as its MIC value, whereas the others had MBIC values that exceeded those of MICs by two to four times, and the MBECs were about two times higher than the MBICs (Table 2 and Supplementary Figure S2). The antimicrobial assay results suggest that temporin-FLa has significantly enhanced bactericidal and biofilm-inhibiting effects, whereas the effects of the other analogue were relatively weak.

The quantitative biofilm formation assay also tested the biofilm’s forming activity after being treated with peptides. The peptides at high concentration could completely inhibit the bacterial aggregation and forming the biofilm, while the 1/2 × MBIC peptides treatment represented moderate biofilm formation capabilities. Besides temporin-FL, the bacteria treated with the 1/4 × MBIC of other peptides showed strong biofilm formation activity. The results (Table 2) indicated that the peptides would show complete biofilm formation inhibiting capabilities above MBIC value, while the decreased peptide would deplete the antibiofilm effect.

**FIGURE 4:**
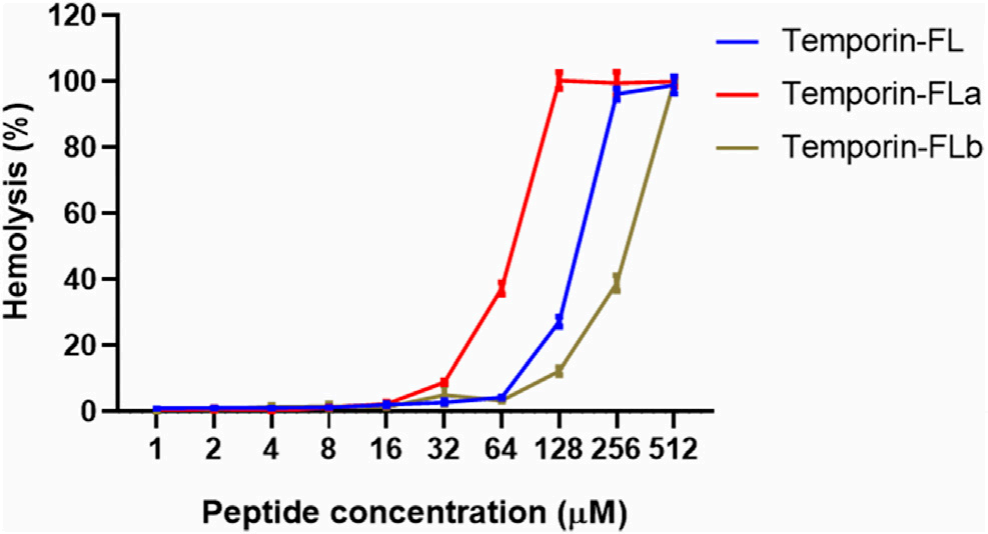
Hemolytic activity of temporin-FL, temporin-FLa and temporin-FLb. The error bar indicates the Standard Error Mean (±SEM) of three replicates.

Temporin-FL and its two analogues showed different degrees of hemolytic activity (Figure 4). The HC_50_ value of temporin-FLb approximately doubled than parent peptide, whereas that of temporin-FLa was the lowest (74.02 μM). The therapeutic index (TI) is a ratio of HC_50_ to the geometric mean of MICs, which characterizes the AMP’s selectivity toward microbial and mammalian cell membranes. The therapeutic indexes of each of the two analogues were two to three times higher than that of the native peptide, and these improved TI values revealed a broader therapeutic window for the two truncated analogues.

**FIGURE 5:**
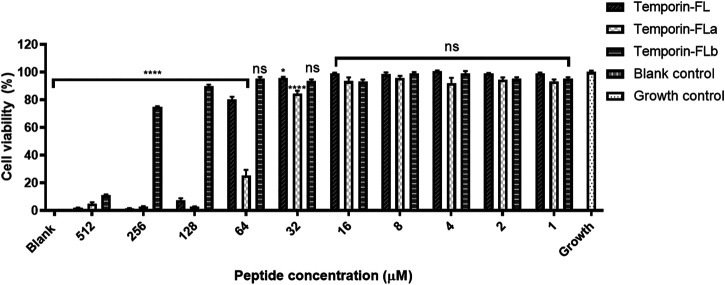
Cell viability on HaCaT cell line of temporin-FL, temporin-FLa and temporin-FLb. The error bar indicates the Standard Error Mean (±SEM) of five replicates. *p < 0.05; ****p < 0.0001, comparing with the growth group.

The cell viability of human keratinocyte cell line HaCaT was measured by the CCK-8 method, the results are shown in Table 2 and Figure 5 indicated that temporin-FL and temporin-FLa have relatively strong cytotoxic effect than temporin-FLb, the IC_50_ values range from 375.1 to 46.62 μM. The cell cytotoxicity of three AMPs corresponded with their antimicrobial activity and hemolytic effect, and their IC_50_ values were much higher than their MIC values. The weak cell growth inhibition on temporin-FL occurred with the dosages as 32 μM, which was equal to its MIC value, while the other peptide were nontoxic towards HaCaT at MICs. The former experiments proved that the two derivatives of temporin-FL had optimized biological activity because they could exert antibacterial activity at the concentration without any cytotoxicity.

### Observation of MRSA Biofilms and the Biofilm Disrupting Potency of Synthesized Peptides Using Scanning Electron Microscopy

**FIGURE 6:**
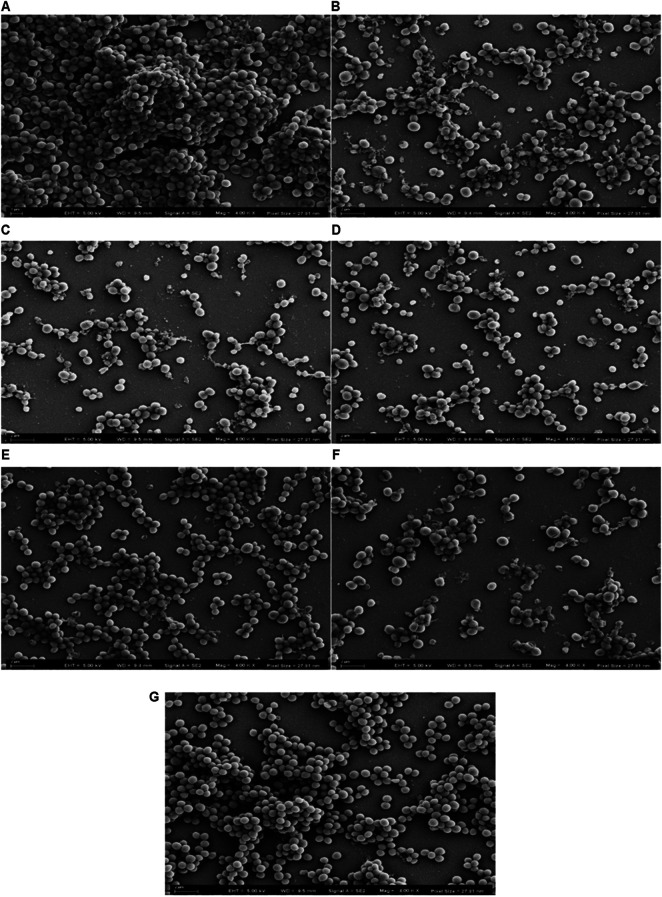
Scanning Electron Microscopy images of MRSA biofilms treated with AMPs, the pixel sizes were set as 27.91 nm. Micrograph of normal MRSA biofilms **(A)**; Biofilms treated with temporin-FL at 4 × MBEC **(B)**; Biofilms treated with temporin-FLa at 4 × MBEC **(C)**; Biofilms treated with temporin-FLb at 4 × MBEC **(D)**; Biofilms treated with temporin-FL at 1 × MBEC **(E)**; Biofilms treated with temporin-FL at 1 × MBEC **(F)**; Biofilms treated with temporin-FL at 1 × MBEC **(G)**.

SEM was used to evaluate biofilm disrupting potency based on clear and detailed observations of the formation of MRSA (ATCC 43300) biofilm treated with three peptides. The regular MRSA biofilm was depicted in Figure 6A, which showed that MRSA formed intensive and abundant aggregations, and the cells maintained their round shape. The results displayed in Figure 6 reveal that all of the tested peptides exhibited potency relating to the eradication of MRSA biofilm at the high concentration (4 × MBEC); the biofilm was disrupted as a result of a drastic decrease in the number of cells and the cell surfaces shrank to varying degrees showed in Figures 6B,C,D. Temporin-FLa exerted superior activity against MRSA biofilms (Figure 6C). However, at a low peptide concentration (1 × MBEC), the microscopy of the biofilm revealed that the tested AMPs caused a slight amount of disruption of MRSA biofilms, and the membrane surfaces of some bacteria were observed to be crumpled and rough (Figures 6E–G). Although all of the AMPs at the low concentration were associated with a reduction of cell colonies, the tested AMPs showed various degrees of biofilm disruption. The parent peptide possessed the moderate bacterial colonies reduction (Figure 6E), temporin-FLb, despite lacking the ability to disrupt membranes (Figure 6G), possessed the capability of inhibiting the formation and aggregation of biofilms. By contrast, temporin-FLa had the greatest effect in reducing the number of bacterial colonies and disrupting cell membranes (Figure 6F). These findings are consistent with the data obtained from the antibacterial assay.

### MRSA Bacterial Membrane Permeability

**FIGURE 7:**
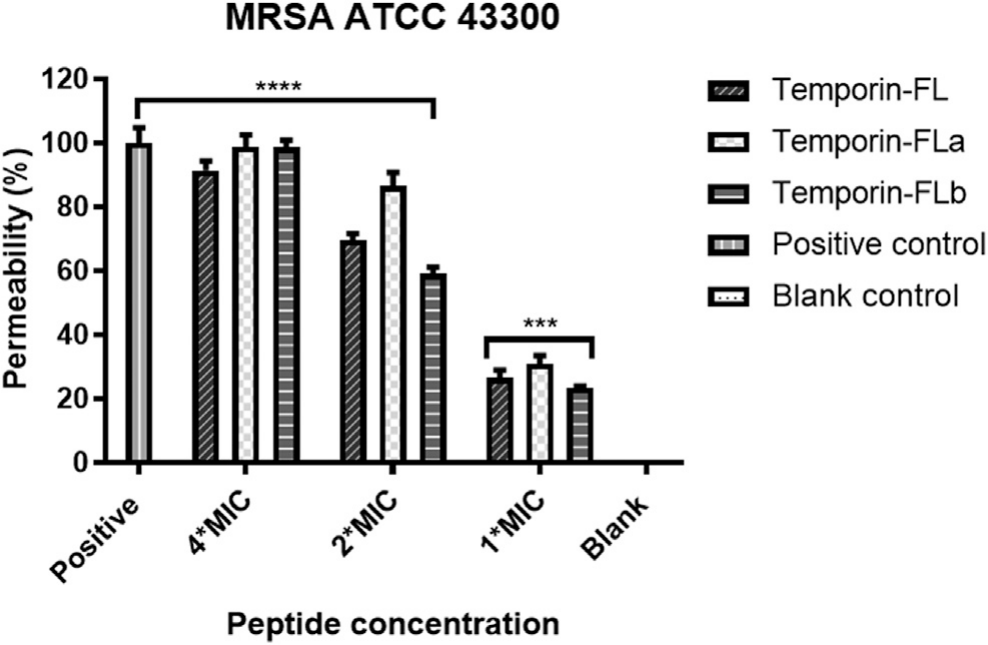
Bacterial membrane permeability against MRSA ATCC 43300. The error bar indicates the Standard Error Mean (±SEM) of five replicates. ***p < 0.001; ****p < 0.0001, comparing with the blank group.

The positively charged AMPs interacted with the negatively charged bacterial outer membranes, which were disrupted as a result of the electrostatic force. SYTOX Green dye binds with nucleic acids when the bacterial outer membrane is disrupted, leading to an increase in measurable fluorescence. The results showed in Figure 7 reveal that temporin-FL and its two derivatives exert membrane permeabilizing efficiency in a dose-dependent manner. Compared with the native peptide, the derived temporin-FLa demonstrated the highest fluorescent intensity during a 3-h incubation period at 4 × MIC, thus demonstrating the significantly enhanced ability of temporin-FLa to disrupt bacterial membranes. On the other hand, the permeabilization potential of temporin-FL and temporin-FLb was relatively weak compared to temporin-FLa at high concentrations, whereas the three peptides showed equal permeabilizing effect at low concentrations on tested MRSA bacterial strains. The membrane permeability capabilities of peptides were consistent with the results of the MIC determination assay shown in Table 2.

### MRSA Bacterial Membrane Depolarization

**FIGURE 8:**
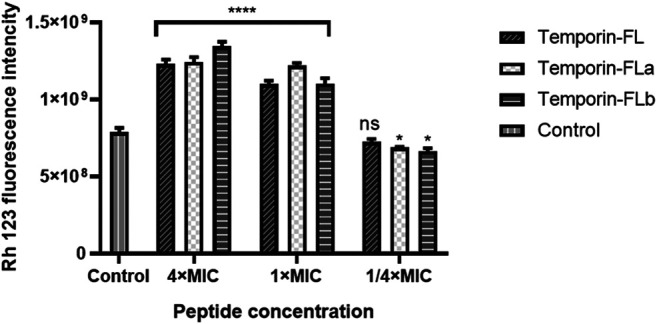
Bacterial membrane depolarization measurement of MRSA ATCC 43300. The error bar indicates the Standard Error Mean (±SEM) of three replicates. *p < 0.05; ****p < 0.0001, comparing with the control group.

The over-dosed fluorescence probe (Rh123) was added to the bacteria and peptide mixture to detect bacterial membrane depolarization. As the membrane was complete, the Rh123 would be incorporated into bacteria and cause the reduction of fluorescence intensity. The fluorescence intensity related to peptides bacterial membrane depolarizing capability in a dose-dependent manner. As the results showed in Figure 8, a rapid fluorescence intensity increase was observed when the concentrations of the peptides were greater or equal to the MICs, which indicated that the positively charged peptides would affect cytoplasmic membrane integrity by membrane depolarization and block bacteria intake of Rh 123. However, the low decrease of fluorescence intensity also appeared in the low concentration group, which might be resulted from the membrane permeability effect of peptides.

## Discussion

Microbial infections are causing increased concern because they induce refractory diseases and complications such as diabetic foot ulcers, urinary tract infections, and cystic fibrosis (Spencer et al., 2013; Bezzerri et al., 2014; Zhao et al., 2020). Currently, antibiotics are the dominant therapy for treating microbial infections. However, epidemiological studies have shown that the global burden of antibiotic resistance is increasing (Cohen, 1992). MRSA is a major pathogen responsible for antibiotic-resistant bacterial infections. Since 1939, the natural defence system of amphibian skin secretions has been explored as a potential natural antimicrobial drug that could economically and effectively resolve this issue (Bahar and Ren, 2013). AMPs have emerged as a novel therapy for treating infections that could replace conventional antibiotics on account of their broad-spectrum antibiosis and lower incidence of drug resistance (Ageitos et al., 2017; Sierra et al., 2017).

Temporin is the smallest peptide family among naturally derived AMPs. Temporin modifications performed within previous studies have mostly involved the replacement of amino acids (Xie et al., 2019), the addition of other amino acids (Avitabile et al., 2013; Bezzerri et al., 2014), and terminal adjustment, entailing the attachment of a small and unique amino acid sequence to the terminal of the original AMPs (Sang et al., 2018). Alterations of the primary structures of temporins significantly affect their bioactivity. The truncation of amino acids in the AMP sequence has emerged as a key technique used for AMP modification. Rational truncation of amino acids has a positive effect of reducing hemolysis while maintaining or partly impairing its bioactivity (Lv et al., 2014; Ying et al., 2019; He et al., 2020; Solstad et al., 2020). However, studies on temporin truncation are rare.

In this study, we identified and characterized a novel AMP, temporin-FL, obtained from the skin secretion of the dark-spotted frog, analyzed its physiochemical properties and characterized its bioactivities. Temporin-FL was found to possess broad-spectrum antibacterial and antifungal activities, while its hemolytic effect was remarkable. We intentionally designed two novel temporin-FL analogues to explore how truncation of the amino acid sequence affects the structural-functional characteristics of the temporin family. The removal of the 10th serine residue on the original amino acid sequence significantly disrupted the hydrophilic face of temporin-FL. The increased hydrophobicity led to the significantly enhanced antimicrobial activity of the novel temporin-FLa derivate. This finding is in agreement with that of previous studies on the positive correlation between alteration of hydrophobicity in peptides and their bactericidal potency (Bahar and Ren, 2013; Tan et al., 2018). Most AMPs contain over half of the hydrophobic residues among their amino acid sequences, and the increased hydrophobicity modulates the selectivity of AMP’s interactions with membranes (Wieprecht et al., 1997; Pasupuleti et al., 2012; Bahar and Ren, 2013). Previous studies have confirmed the existence of an optimum hydrophobic window for modulating antimicrobial activity, with enhanced efficiency being correlated with an increase in hydrophobic residues (Chen et al., 2007).

Moreover, alteration of the primary sequence induced a drastic change in the amphipathicity (characterized mathematically by the hydrophobic moment [μH]). Temporin-FLa demonstrated an enhanced value for the hydrophobic moment compared with that of the parent peptide, which could lead to greater hemolytic activity. Previous studies have shown that attenuated amphipathicity restricts the hemolysis of AMPs (Fernández-Vidal et al., 2007; Hollmann et al., 2016). Because the design of temporin-FLb entailed the removal of the fourth leucine residue of temporin-FLa, this analogue showed reduced amphipathicity and while not cytotoxic to mammalian erythrocytes, demonstrated lower antimicrobial activity. The truncated 10th serine residue of temporin-FL increased the hydrophobicity of temporin-FLb but did not enhance its antimicrobial effect, indicating that the suppressed microbial membrane-disrupting potency was mostly affected by reduced hydrophobicity. These findings suggest that the hydrophobicity and amphipathicity of AMPs control the performance of their activities relating to cell membranes and that the extent of peptide’s penetration into cell membranes depends on their hydrophobicity (Pasupuleti et al., 2012). Amphipathicity has a stronger effect on peptide-membrane interfacial binding (Fernández-Vidal et al., 2007) and is also associated with the formation of the helicity structure. However, it remains unclear which of these physicochemical properties has the most influence on the antimicrobial effect.

The results of the secondary structure analysis revealed that the three peptides formed a coli structure in a water environment but underwent a conformational transformation into an α-helical structure in a mimicked membrane environment. The temporins adopt extended or unstructured conformers in other environments, this conformational phase changes would prevent indiscriminate membranolytic activity and lower the host cell toxicity (Travkova et al., 2017). A previous study about TFE as a peptide stabilizes cosolvent indicated that TFE could help helical peptides maintain their secondary structure by promoting intramolecular hydrogen bond formation and peptides aggregation (Chaubey et al., 2020), therefore reflects the mechanisms of peptide-membrane interaction when the AMPs contact with pathogenic microorganisms. This phenomenon has also been reported in previous studies of temporins (Mangoni, 2006; Sang et al., 2018). The observed AMPs were positively charged by lysine residue located on the hydrophilic side of the predicted helical wheels (Figure 3), the former study also indicates that the lysine residue on peptides is often at the lipid/water interface, while other hydrophobic residues were located at the opposite side, demonstrating a classical model of an α-helical peptide (Travkova et al., 2017; Zhong et al., 2019). The membrane-active peptides exhibited amphiphilic properties, which are usually performed as helical structures. Using a combination of charged and hydrophobic residues, the amphipathic helical structure binds to the microbial membrane interfaces with the polar surface facing the membrane hydrophilic residues, while the non-polar surface faces the hydrocarbon interior of the bilayer (Dathe et al., 1996; Fernández-Vidal et al., 2007).

The helicity of AMPs was shown to have crucial effects on transmembrane pore formation related to lipid–peptide interactions. Cytotoxic temporins assume a helical structure when binding with the zwitterionic membrane, and nontoxic peptides are unable to form a complete helical structure in zwitterionic lipid vesicles (Kumari et al., 2020). The direct correlation between peptide helicity and toxicity toward normal cells was reported previously (Mangoni et al., 2016). These findings concur with our experimental results of the antimicrobial and cytotoxicity evaluation assay of the three examined peptides, as temporin-FLb, which had a radically disrupted helical structure, evidenced lower antimicrobial efficiency than the original peptide. However, the decreased helicity in temporin-FLa subtly influenced its bioactivity, which could be attributed to the considerable change in hydrophobicity. The parent peptide had the most complete helical structure in lipid simulating envirnoment, thus the cytotoxicity towards HaCaT cell line was close to its MIC values. In sum, microbial inhibition and eradication are complex processes, and the physiochemical properties of AMPs and membrane lipids affect AMP’s antimicrobial activity. The interconnections of influential internal factors require further study.

The results of the antimicrobial and cytotoxicity evaluation assay showed that all three peptides exhibited potent activity against Gram-positive bacteria and fungi but were unable to eliminate *E. coli*, which is a Gram-negative bacterium. The antimicrobial activity of temporin-FLa was found to be stronger than that of the other two peptides. Previous studies revealed a similar trend relating to the antimicrobial potency of temporins against Gram-positive bacteria and fungi, but temporins were insensitive to Gram-negative bacteria (Mishra et al., 2018). The MICs of three AMPs against Gram-positive bacterial strains ranged from 4 to 64 μM, and those against fungi ranged between 8 and 32 μM. These results concur with the findings of another study on temporin-1KM, with a similar primary structure to temporin-FL, which demonstrated strong activity against *S. aureus* and *C. albicans*, with MICs of approximately 4 and 8 μM, respectively (Song et al., 2013). The diverse antimicrobial effects of temporins on different bacteria can be largely attributed to the cell envelopes of the latter. Gram-negative bacteria have outer membranes containing phospholipids, lipopolysaccharide (LPS), and porins (Silhavy et al., 2010). The LPS and porins of Gram-negative bacteria have essential barrier functions against hydrophobic molecules (Nikaido, 2003; Silhavy et al., 2010). Many peptides in the temporin family have antifungal properties, with the α-helical structure being the likely cause of disruption of fungal membranes (Wade et al., 2001; Rollins-Smith et al., 2003). In the hemolysis and cell viability assay, temporin-FLa shows a enhanced hemolytic and cytotoxicity effect, which is probably related to the increased hydrophobicity reduced the peptide selectivity towards membranes (Chen et al., 2007; Takahashi et al., 2010). However, the therapeutic index indicates temporin-FLa has improved therapeutic window, and the concentrations causing half of erythrocytes collapse and HaCaT cell disruption are much higher than the bactericidal concentration, which makes temporin-FLa is fit for the effective application under low concentration. Temporin-FLb also shows optimized bioactivity by drastically reduced its cytotoxicity and possesses increased TI value.

Antibiotic-resistant bacterial strains are the main cause of refractory infections. Therefore, two representative MRSA strains were tested to investigate the antimicrobial mechanisms of synthesized AMPs. The results of the membrane permeability assay indicated that the tested peptides exhibited bacterial membrane-permeable activity, and the efficiency of permeability was positively correlated with peptide concentration. Temporin-FLa had the greatest permeability among the three peptides and temporin-FL possessed moderate permeabilizing activity, while the potency of temporin-FLb was the lowest except for the 4 × MIC concentration. The dramatic increase of temporin-FLb at high concentrations could be linked to the bacterial damage caused by increased osmotic pressure. The driving force behind the membrane disruption activity of temporins is the binding of the AMP’s helical structure and microbial membranes that occurs in a barrel-stave manner, leaving behind a hole in the membrane, the “barrel-stave” model required not only the electrostatic interaction of AMPs and lipids but also the aggregation of the peptides. For example, temporin L and temporin B were found to aggregate and form the toxic oligomers in the membrane, then these oligomers are subsequently converted to amyloid-type fibres that involve acidic phospholipids-induced conformational changes (Mahalka and Kinnunen, 2009), the driven forces for peptide aggregation might be related to hydrogen bond and hydrophobic interaction (Wang et al., 2016; Travkova et al., 2017). Thus, intracellular molecules are able to pass through the membrane, ultimately leading to cell death (Mangoni et al., 2004; Mangoni, 2006). The membrane penetrating AMPs have broad application prospects, the previous researches found their usage as food preservatives (Chikindas et al., 2018; Aymerich et al., 2019), the antimicrobial surgical dressing products to prevent wound infections (Lin et al., 2019; Chen et al., 2021), and even developed into a novel preparation like hydrogel for the prophylaxis of bacterial vaginosis (Aymerich et al., 2019).

Rhodamine123 is a membrane potential sensitive fluorescent dye that is always used in measuring membrane depolarization (Chakraborty et al., 2012). The Rh 123 dye would lose fluorescence intensity when the membrane was polarized while the depolarized membrane would block the incorporation of the dye into the cytoplasmic membrane thus highly increased the fluorescence intensity. The results of measuring fluorescence intensity indicated that the three AMPs possessed membrane depolarization ability in a dose-dependent manner, the rapid increase of Rh 123 fluorescence signal was observed in high concentrations of peptides. The membrane depolarization was closely related to AMPs bactericidal activity, the inner mechanism was correlated with K^+^ ions release (Silverman et al., 2003).

Bacterial biofilms are formed by surface colonies of microbes that produce a matrix to protect the bacterial community (López et al., 2010), thus impeding regular anti-infection therapy. Consequently, novel anti-biofilm agents are urgently required. All three tested AMPs revealed antibiofilm activity in a dose-dependent inhibitory manner, and their antibiofilm potency corresponded to their antimicrobial strength, indicating that the AMPs eradicate both planktonic and biofilm-associated MRSA strains. SEM observations revealed that incubation of the peptides with MRSA biofilm caused shrinkage of cell surfaces and biofilm disruption, as the initial formation and further association of the bacterial biofilm were blocked. The quantitative biofilm formation assay also revealed that three AMPs could block biofilm formation at high concentrations and lose their potency in lower concentrations below MBIC. The antibiofilm mechanisms against MRSA might be related to disrupting or degrading membrane potential of biofilm embedded cells or degrading biofilm matrix (Yasir et al., 2018). The AMPs designed for this study are attractive candidates and the leading compounds for use in the treatment of microbial infections in cases where the abuse of antibiotics leads to the development of bacterial strains that are resistant to antibiotics.

## Conclusion

In conclusion, we identified a novel AMP from frog skin secretion and designed two truncated analogues with different structures. The modification of the peptide’s helicity and hydrophobicity could alter their bioactivity. Temporin-FLa demonstrated improved hydrophobicity and a higher value for the hydrophobic moment, whereas temporin-FLb possessed reduced helicity and amphipathicity. Both analogues exhibited an enhanced therapeutic index in relation to the tested microbes compared with the parent peptide. All of the tested peptides mainly conducted α-helix when interacting with microbial membranes, and they also demonstrated the ability to bind to membrane lipids with their positive net charge, inducing membrane permeability. The three peptides also cause membrane depolarization in a dose-dependent manner, which further proved that they exert antimicrobial effects through perturbing the membrane integrity. Moreover, all of the studied AMPs demonstrated antibiofilm activity against MRSA, which is a key prerequisite for the development of AMP microbial agents. The derived peptides, characterized by a significantly enhanced therapeutic index, showed an improved active window comparing with the native peptide, indicating that they are promising anti-infection candidates in addition to being a novel insight for small AMP modification.

## Data Availability

The datasets presented in this study can be found in online repositories. The names of the repository/repositories and accession number(s) can be found in the article/Supplementary Material.

## Supplementary Material

Supplementary Figure S1 Inhibitory effects of Temporin-FL, Temporin-FLa and Temporin-FLb against *S. aureus* **(A)**, MRSA (NCTC 12493) **(B)**, MRSA (ATCC 43300) **(C)**, *E. coli* **(D)**, *C. albicans* **(E)** in a range of concentrations from 512 to 1 μM, the inhibition rate fluctuating in high concentrations for peptide precipitating at high concentrations. Data represent means ± SEM.

Supplementary Figure S2 Eradication effects of Temporin-FL, Temporin-FLa and Temporin-FLb against the biofilm formed by MRSA NCTC 12493 **(A)** and MRSA ATCC 43300 **(B)**. Data represent means ± SEM.

Supplementary Figure S3 The helical wheels of the parent peptide **(A)** and derivatives 1–6 **(B–G)**, the yellow colour represents nonpolar residues, the purple colour indicates polar residues, the blue colour represents charged residues, the grey and green indicated special residues glycine and proline.

Supplementary Table S1 The potential peptide derivative and the related parameters.
