# Supplementary figures and images for: Bioevaluation and Targeted Modification of Temporin-FL From the Skin Secretion of Dark-Spotted Frog (Pelophylax nigromaculatus)
Source: Front Mol Biosci. 2021 Oct 19;8:707013. doi: 10.3389/fmolb.2021.707013 (PMC8560897; doi:10.3389/fmolb.2021.707013)

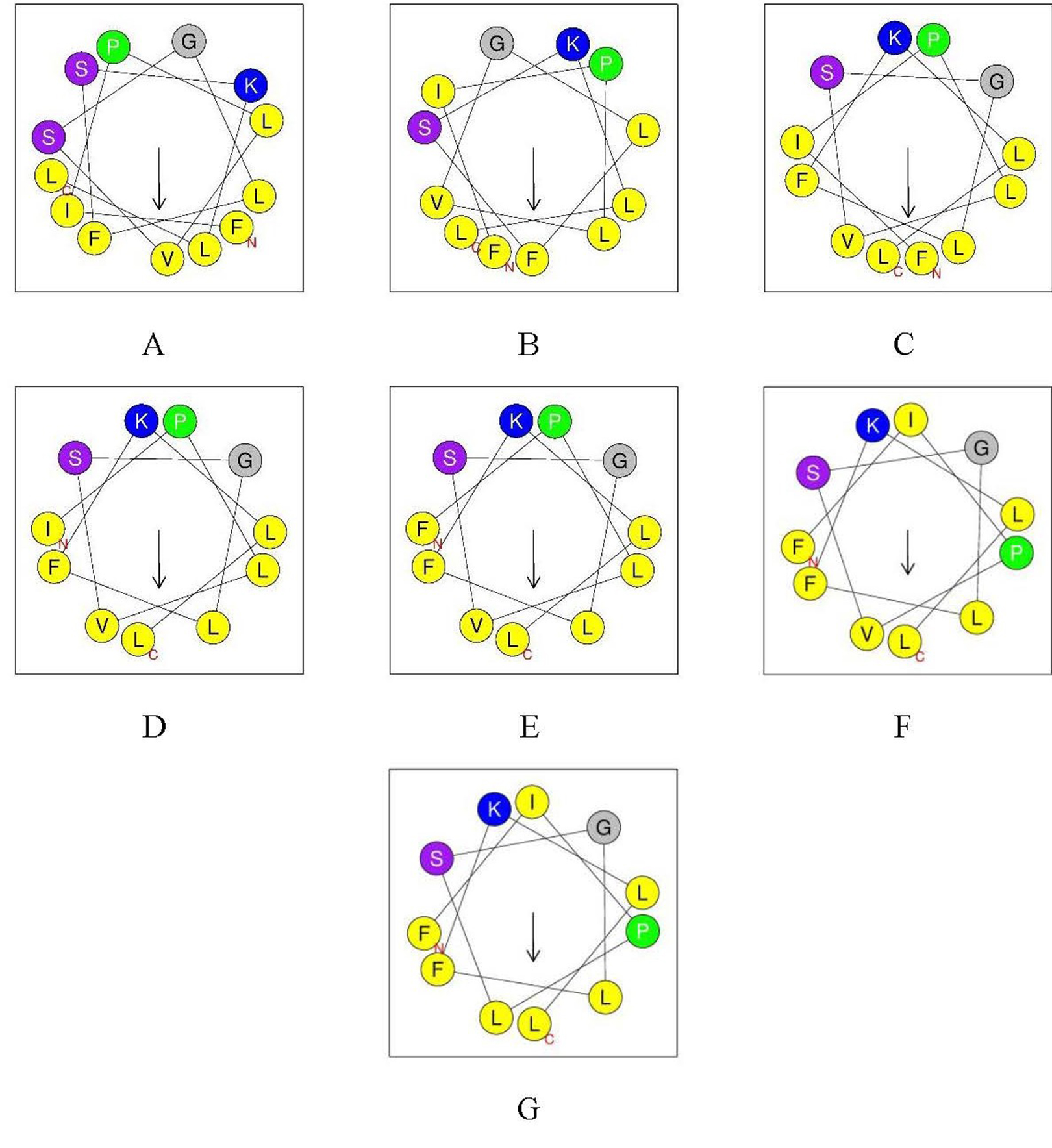

Supplement: Supplementary file 1 [file Image3.jpg]

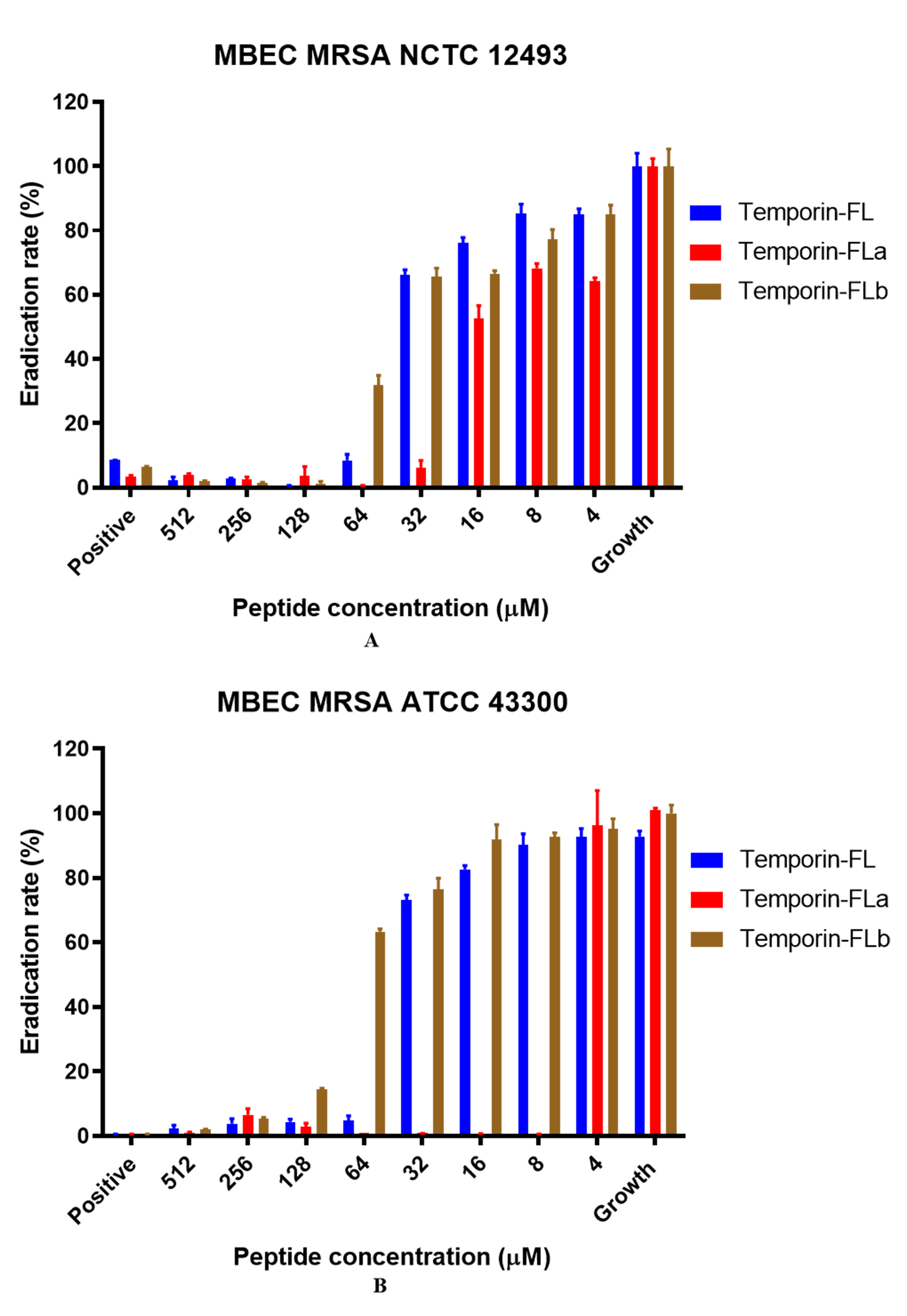

Supplement: Supplementary file 3 [file Image2.TIF]

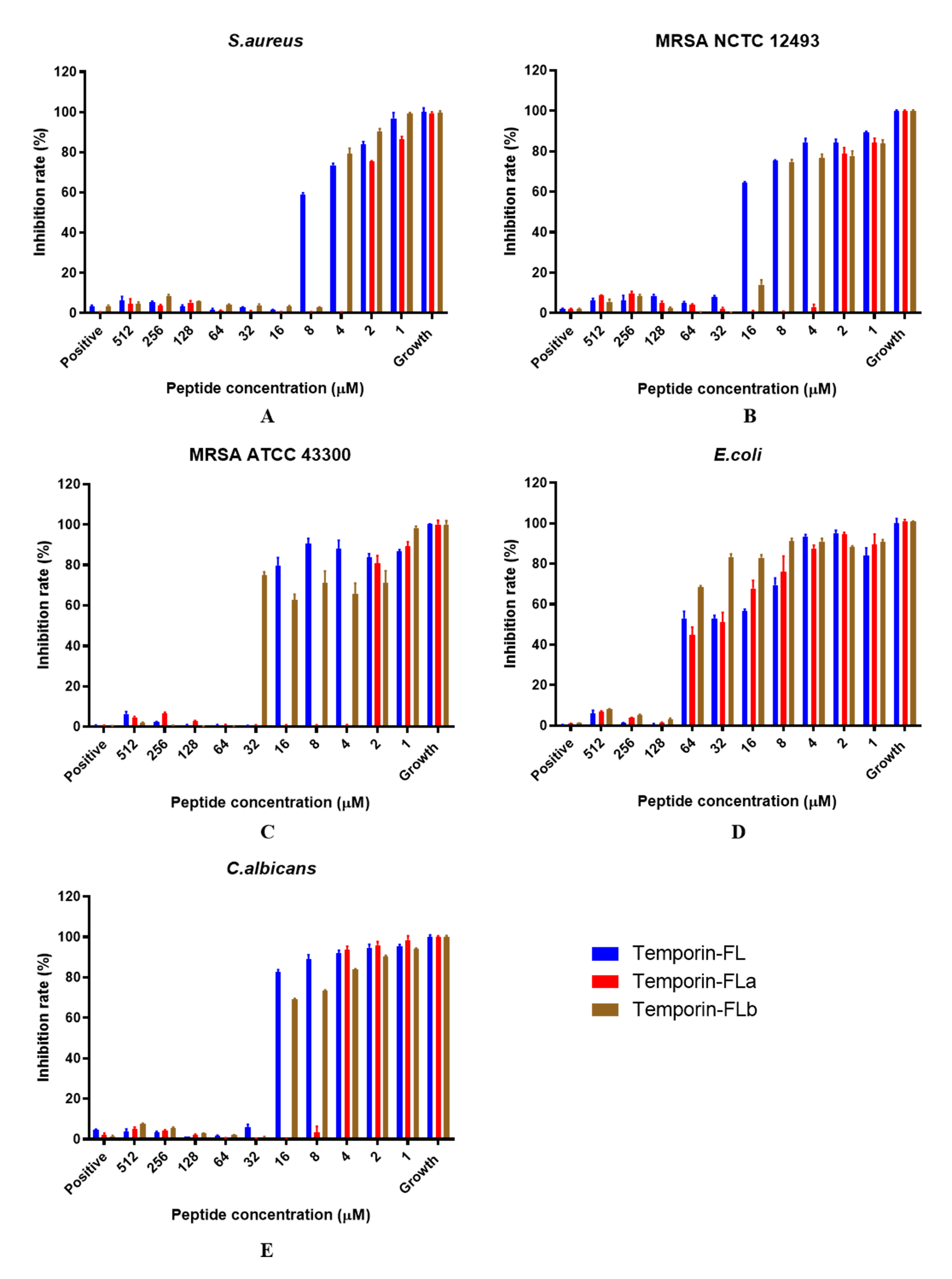

Supplement: Supplementary file 4 [file Image1.TIF]
