# Supplementary material for: Bioevaluation and Targeted Modification of Temporin-FL From the Skin Secretion of Dark-Spotted Frog (Pelophylax nigromaculatus)
Source: Front Mol Biosci. 2021 Oct 19;8:707013. doi: 10.3389/fmolb.2021.707013 (PMC8560897; doi:10.3389/fmolb.2021.707013)
Supplement: Supplementary file 2 [file Table1.DOCX]

Table S1. The potential peptide derivative and the related parameters

| Name | Sequence | Hydrophobicity | Hydrophobic moment | AMP probability in SVM model | AMP probability in RF model |
| --- | --- | --- | --- | --- | --- |
| Temporin-FL | FIPLVSGLFSKLL-NH2 | 1.004 | 0.553 | 0.961 | 0.967 |
| Derivative1 | FIPLV_GLFSKLL-NH2 | 1.091 | 0.561 | 0.959 | 0.97 |
| Derivative2/Temporin-FLa | FIPLVSGLF_KLL-NH2 | 1.091 | 0.622 | 0.966 | 0.97 |
| Derivative3 | _IPLVSGLF_KLL-NH2 | 1.027 | 0.517 | 0.879 | 0.901 |
| Derivative4 | F_PLVSGLF_KLL-NH2 | 1.026 | 0.517 | 0.942 | 0.8165 |
| Derivative5/Temporin-FLb | FIP_VSGLF_KLL-NH2 | 1.035 | 0.394 | 0.923 | 0.9005 |
| Derivative6 | FIPL_SGLF_KLL-NH2 | 1.079 | 0.435 | 0.962 | 0.9165 |
